# An automated approach to identify sarcasm in low-resource language

**DOI:** 10.1371/journal.pone.0307186

**Published:** 2024-12-05

**Authors:** Shumaila Khan, Iqbal Qasim, Wahab Khan, Aurangzeb Khan, Javed Ali Khan, Ayman Qahmash, Yazeed Yasin Ghadi

**Affiliations:** 1 Institute of CS & IT, University of Science & Technology, Bannu, Pakistan; 2 Department of Computer Science, School of Physics, Engineering & Computer Science, University of Hertfordshire, Hatfield, United Kingdom; 3 Department of Informatics and Computer Systems, King Khalid University, Abha, Saudi Arabia; 4 Department of Computer Science, Al Ain University, Al Ain, UAE; University of Kurdistan Hewler, IRAQ

## Abstract

Sarcasm detection has emerged due to its applicability in natural language processing (NLP) but lacks substantial exploration in low-resource languages like Urdu, Arabic, Pashto, and Roman-Urdu. While fewer studies identifying sarcasm have focused on low-resource languages, most of the work is in English. This research addresses the gap by exploring the efficacy of diverse machine learning (ML) algorithms in identifying sarcasm in Urdu. The scarcity of annotated datasets for low-resource language becomes a challenge. To overcome the challenge, we curated and released a comparatively large dataset named Urdu Sarcastic Tweets (UST) Dataset, comprising user-generated comments from 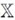 (former Twitter). Automatic sarcasm detection in text involves using computational methods to determine if a given statement is intended to be sarcastic. However, this task is challenging due to the influence of the user’s behavior and attitude and their expression of emotions. To address this challenge, we employ various baseline ML classifiers to evaluate their effectiveness in detecting sarcasm in low-resource languages. The primary models evaluated in this study are support vector machine (SVM), decision tree (DT), K-Nearest Neighbor Classifier (K-NN), linear regression (LR), random forest (RF), Naïve Bayes (NB), and XGBoost. Our study’s assessment involved validating the performance of these ML classifiers on two distinct datasets—the Tanz-Indicator and the UST dataset. The SVM classifier consistently outperformed other ML models with an accuracy of 0.85 across various experimental setups. This research underscores the importance of tailored sarcasm detection approaches to accommodate specific linguistic characteristics in low-resource languages, paving the way for future investigations. By providing open access to the UST dataset, we encourage its use as a benchmark for sarcasm detection research in similar linguistic contexts.

## 1 Introduction

Social media platforms have transformed the public communication style, prompting research to shift from information retrieval to opinion mining. As users adopt informal and non-standard language in their online interactions, such linguistic nuances play a crucial role in sentiment analysis. The bulk of data on social media has led to several challenges in extracting public opinion from user-generated content, automatically identifying and summarizing the polarity of a document, and detecting irony, hate speech, and sarcasm, which need to be explored. Sarcasm has been studied in various fields, including linguistics, psychology, and communication studies. In linguistics, researchers have analyzed the linguistic features of sarcasm, such as intonation, stress, and prosody, to understand how it is conveyed in speech [1, 2].

Sarcasm has been researched as a form of verbal aggressiveness in psychology and linked to personality qualities, including cynicism and hostility [3]. However, automatic sarcasm detection in the text has been significant in computing areas such as natural language processing [4], enhancing sentiment analysis, avoiding miscommunication, and improving human-robot interaction [5–7]. Sarcasm can be found in numerous languages, and its manifestation and comprehension vary depending on the cultural and linguistic backdrop. For example, sarcasm is commonly used in English and is often characterized by a change in tone or emphasis. Automatic sarcasm detection models must consider cultural and linguistic differences to accurately detect sarcasm in different languages. Researchers have developed sarcasm detection models for several languages, including Hindi [8], Indonesian [9], Dutch [10], English [11, 12], and Filipino [13]. At the same time, low-resource languages such as Urdu, Roman-Urdu, Pashto, Punjabi, and Hindi are poor resource languages and need further development [14, 15].

The Urdu language also has several words that refer to the act of sarcasm or its related concepts, including طنزیہ (Tanzia): sarcasm, مذاق (Mazaq, joke or jest), "Istihza" (استہزاء, mockery), "Latifa"(مضحکہ خیز, Humour), "Tabasum" (تبسم,). These words can also be used in different contexts and may have slightly different connotations depending on the situation. Table 1 illustrates examples of sarcastic tweets along with their corresponding English and Roman-Urdu translations each sentence presents a sarcastic remark. The example text overall is positive, but if we consider the emoticon with the text, then the impact of the ruling becomes negative and is marked as sarcastic. There are also exaggerations, punctuation marks, emojis, user behavior, and tone, which make the text ambiguous and sarcastic.

**Table 1:** Examples of sarcastic tweets.

Urdu Text	Roman-Urdu	English
اگر جماعتیں کم پڑ گئیں ہوں تو پڑوس سے کانگرس اور بی جے پی کو بھی بلوا لیں 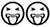	Agr jamatian kam pard gai hn to pardos sy congreous aur PJP ko bhi bula lain 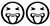	If the number of Jamaat-e-Islami members has decreased, then invite Congress and BJP from the neighboring areas as well 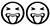 "
**کراچی کے موسم کو بھی ہارٹ اٹیک آیا ہوا ہے صبح سردی دن میں گرمی رات میں سردی اور گرمی۔۔۔۔۔۔۔ 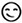 **	karachi k mosam ko b heart attack aya hoa hy, subah sardi, din mai garmi aur rat mai sardi aur garmi۔۔۔۔۔ 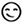	"The weather in Karachi is also experiencing a heart attack, with cold in the mornings and heat in the afternoons and nights. 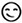 "
آلو200روپےدرجن 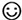 انڈے200کلو 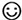 آٹا600روپےلیٹر 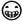 چینی 100 روپےمربع فٹ 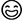 سونا1لاکھ روپےکلومیڑ 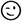 سائنسدان کا نام بتائیں؟ 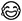	Aalu 200 rupy darjan 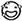 anday 200 kilo 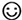 Aata 600 rupy litr 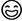 cheni 100 rupy muraba foot 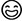 , soona aik lakh rupy km 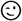 , sciencedaan ka naam batian?. . . 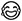	"Potatoes cost 200 rupees per dozen 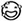 Eggs are 200 rupees per kilogram 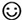 Flour is 600 rupees per liter 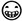 Sugar is 100 rupees per square foot 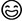 Gold costs 1 lakh rupees per Kilometre 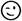 Can you name the scientist? 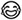 "
کیپٹن صفدر کی گرفتاری نے ثابت کر دیا کہ۔ غریب آدمی کو پہلے تو موقع نہیں ملتا، موقع مل جائے تو جگہ نہیں ملتی اور اگر موقع اور جگہ دونوں مل جائیں تو چھاپہ پڑ جاتا ہے۔ 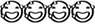	Captain Safdar ki giraftari ny sabit kar dia k ghareeb admi ko pahly moqa ni milta, moqa mil jay to jaga ni milti aur agr moqa aur jaga dono mil jain to chapa par jata hy, 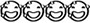	"Captain Safdar’s arrest has proven that a poor man does not get a chance at first, and even if he gets an opportunity, he does not get the right place. Moreover, if he gets both opportunity and the right place, then a raid occurs. 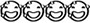 "

Automatic sarcasm detection in text involves using computational methods to determine if a given statement is intended to be sarcastic. However, this task is challenging due to the influence of the user’s behaviour and attitude and their expression of emotions. This is especially true in languages with complex morphology, like Urdu, where contextual nuances and cultural references can further complicate the task of sarcasm identification. Detecting sarcasm in Urdu aims to enhance sentiment analysis research.

This paper proposes an ML-based approach to identify sarcasm in user comments written in Urdu. By conducting a comparative study of various state-of-the-art ML models, we aim to recognize the most effective model for sarcasm detection in Urdu, which can serve as a benchmark for future studies. To assess the efficacy of the proposed framework, we conducted a thorough comparison with a baseline model Tanz-Indicator [16]. These methodologies have been developed to discern and classify sarcasm within Urdu using a supervised ML approach. The comparison involves a comprehensive assessment of the models to identify their strengths, weaknesses, and critical differences, e.g., system accuracy, efficiency, and dataset scalability. The results of the model’s performance demonstrate that the proposed framework exhibits superior effectiveness and accuracy when measured against the baseline. This performance enhancement is evident not only in terms of the model’s predictive capabilities but also in the context of the expanded dataset. These findings collectively contribute to the understanding that the proposed framework offers an advancement over the baseline, making it a compelling choice for applications requiring model performance.

The study focused on classifying the instances of sarcasm in Urdu, as it significantly enhances NLP research and facilitates practical applications over diverse domains. This study addresses the gap in sarcasm identification for low-resource languages, emphasizing Urdu. The development of the Urdu-Sarcastic-Tweets-Dataset (UST) and the evaluation of state-of-the-art machine learning models provide a base for future research and practical applications in SA, human-computer interaction, and social media monitoring. Incorporating advanced ML models including XGBoost and employing N-grams for feature extraction significantly enhance the accuracy of identifying sarcasm in Urdu. By leveraging the strengths of these models and feature extraction techniques, the proposed approach achieves higher precision, recall, and F1-scores than existing approaches. In all these aspects, developing effective sarcasm detection in Urdu aligns with the broader aspiration of NLP research—to bridge language and cultural gaps while fostering more precise, empathetic, and context-aware communication technologies. The main contributions and achievements made in this paper are listed below.

- Developed and curated a novel low-resourced language (Urdu) research dataset comprising user tweets labelled as sarcastic and non-sarcastic.
- Developed a novel grounded theory for low-resource language-based sarcasm detection.
- Developed a truth set for sarcasm detection using a content analysis approach.
- Employed various ML classifiers to recover their performances in detecting sarcasm for low-resourced language using various feature engineering approaches.

Roadmap: The article is structured as follows: Section 2 provides related works and background. Section 3 presents the proposed research approach. Section 4 presents an overview of the dataset’s processing. Section 5 discusses the automated classification of sarcasm in low-resource language. Section 6 presents the overall discussion of this research comprehensively. Finally, we conclude the article in Section 7.

## 2 Related works and background

Several studies have been conducted in sarcasm detection using various approaches, including rule-based, feature-based, and machine-learning-based methods. Researchers in NLP and ML have recently expressed a strong interest in automated sentiment analysis [17, 18], including a Neural recommender system for representative learning [19], an Emotion-semantic-aware model for learner-generated reviews [20], and a multi-labelled carpus for 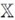 tweets [21]. ML uses supervised and unsupervised classification techniques to identify sarcasm in text. Rule-based approaches rely on handcrafted rules and linguistic patterns to identify sarcastic comments. This study used a comparative analysis of different ML algorithms and an n-gram approach to detecting sarcasm in Urdu.

### 2.1 Sarcasm and sentiment approaches based on English language

Sarcasm detection in English text has gained significance recently due to its prevalence and inherent complexity. Previous research on sarcasm detection was thoroughly reviewed by Eke et al. [22] by analyzing articles written in English. Based on their findings, N-grams and part-of-speech tags (POS) were the most used feature extraction algorithms. Feature representation primarily relied on binary interpretation and word frequencies. The review also highlighted the frequent use of the chi-squared test and information gain (IG) method for feature selection. Various classification techniques were employed, like maximum entropy, naive Bayes, random forests (RF), and support vector machine (SVM). In another study, the author proposes a multi-feature fusion framework consisting of two classification stages. In the first stage, the lexical feature extracted by BoW is used to train five standard classifiers (SVM, DT, KNN, LR, and RF) to predict the sarcastic tendency of a tweet. In the second stage, the sarcastic tendency feature is fused with eight other contextual features to obtain the final prediction using a Random Forest classifier [23]. Also, a pattern-based approach was implemented for sarcasm analysis, utilizing a scoring system and linguistic patterns to evaluate text and distinguish between sarcastic and non-sarcastic expressions [24]. A deep learning technique on the MUStARD Dataset and existing SARC dataset for automatically identifying sarcasm was presented by [25].The results demonstrate that an attention-based long short-term memory (LSTM) architecture achieves the highest F1 score of 60.1.

Similarly, Potamias et al. [26] present a transformer-based approach for detecting irony and sarcasm in text. The researchers leverage the power of transformer models, specifically the BERT architecture, to capture contextual information and semantic relationships within the text. They propose a fine-tuning strategy where the pre-trained BERT model is trained on labelled datasets specifically annotated for irony and sarcasm. A lexicon-based framework for sarcasm detection in Perso-Arabic-scripted Urdu—Tanz-Indicator designed specifically for sarcasm detection in Perso-Arabic Urdu user comments [16]. The framework utilizes a lexicon comprising more than 3000 sarcastic tweets and 100 sarcastic features for experimentation.

Additionally, two ML models are trained and compared with the lexicon-based model. Although the framework is limited to detecting sarcasm in user comments, it cannot be used to detect sarcasm in other types of text, such as news articles or blog posts. For Urdu sarcasm detection, [27] proposes a context-aware approach to enhance sarcasm and sentiment analysis for Urdu, a resource-poor language. The approach incorporates cognitive relationships between words in a sentence to improve the accuracy of sentiment analysis and sarcasm detection. However, they did not explain how the cognitive relationship features are extracted and represented. Furthermore, the study addresses the issue of data scarcity and quality for the Urdu language. The datasets used in the paper are relatively small and may not cover all the possible scenarios and variations of sarcasm and sentiment in Urdu.

### 2.2 Sarcasm detection for low resource languages

Recently, significant progress has been made in Urdu language research. Much research has been done on Urdu sentiment analysis, while Urdu sarcasm is still under research. The research studies have explored the analysis of Urdu through different techniques, encompassing dataset analysis, named entity recognition (NER), analysis of morphology and stemming, identification of stop words, and exploration of concept searching [28]. Moreover, the researchers have utilized various linguistic resources and language pre-processing techniques such as identifying sentence boundaries, tokenizing, part-of-speech tagging, NER, and creating WordNet lexicons to enhance sentiment analysis [29].

A study focused on hyperbolic-based features for sarcasm detection in Hindi tweets [8]. They used interjection words and intensifiers to indicate sarcasm, improving classifiers’ performance in sarcasm classification. However, their study is based only on hyperbolic features, which cannot identify sarcasm sentiment. In their work, Oxana Vitman et al. [30] introduce a model that incorporates contextual, sentimental, and emotional features to capture the dependencies associated with sarcasm. The researchers conducted experiments using four datasets, i.e., SARC/movies, SARC/technology, IAC-V2, and 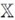 (former Twitter). They employed a deep learning approach consisting of a sarcasm Pre-Trained Transformer (SarcPTT), an Emotion Detection Pre-Trained Transformer (EDPTT) trained on various datasets, a Sentiment Analysis Pre-Trained Transformer (SentAPTT), and a CNN block. Similarly, Liu et al. [31] focus on improving the accuracy of text classification, particularly for Chinese texts, using an adaptive features selection algorithm. They propose three improved feature selection algorithms, including an enhanced CHI square with mutual information (MI), a term frequency-CHI square(TF-CHI) and a term frequency (TF-IDF) algorithms with SVM and NB classifiers. Their results show that the proposed feature selection algorithms improve performance across Chinese new corpora. Liao et al. [32] propose a novel integrated multi-task model for fake news detection that leverages textual and social context features. The model consists of three subtasks: stance detection, veracity prediction, and source reliability estimation.

Singh et al. [33] provide a review of the existing research on sentiment analysis in the Urdu language. The study aims to comprehensively understand the methods and techniques employed in sentiment analysis for Urdu. Another study on developing a sentiment lexicon specifically tailored for sentiment analysis in Urdu is considered a resource-poor language in terms of available linguistic resources [34].

### 2.3 Comparative study

In conclusion, many research approaches have been proposed in the literature for sarcasm detection using English language corpora. However, sarcasm detection for low-resourced languages is still under-researched and needs further focus from the research community. In particular, Urdu language-based sarcasm detection is a relatively new research area in NLP, and a limited number of studies have been conducted in this domain. Therefore, to fill this gap, we proposed a research approach that employs various ML classifiers to identify sarcasm in Roman-Urdu tweets. Unlike English, low-resourced languages are complex to process and challenging to produce generalized results. For this purpose, the proposed approach employs various feature engineering and text-preprocessing approaches to produce comparatively better results.

### 2.4 Characteristics of Urdu

Language plays a pivotal role in interpreting a society’s cultural legacy and emotional nuances. With substantial textual data, automatic content analysis helps us gain insights and understand society. Currently, more than 7,000 languages are used globally. Leading this linguistic diversity, English emerges as the most widely spoken language, boasting a staggering 1.35 billion speakers worldwide, as documented by Ethnologue. Notably, 80% of English speakers use the language as a second, third, or even higher-order language, with a mere two out of every ten native speakers. As the second most widely spoken language worldwide, Mandarin Chinese claims an impressive 1.12 billion speakers. Intriguingly, 921 million individuals wield it as their primary language of expression [35].

Hindi is spoken by around 600 million speakers worldwide. Spanish comes next with 543 million speakers, followed by Arabic with 274 million, and Urdu with 230 million speakers. Languages are written using different sets of symbols (scripts). There are about 294 scripts worldwide; 133 are historical scripts like Egyptian Hieroglyphs and Aztec pictograms, which are not used. However, 161 scripts are still being used today. Languages have exciting aspects that reflect the uniqueness of each culture. Every year, on February 21st, World Mother Language Day is celebrated to honour the diversity of languages worldwide [36, 37]. Latin, employed in over 305 languages globally, which includes English, French, and Spanish, stands out as the most widespread among the 161 active scripts currently present.

Urdu, the native language of Pakistan, is spoken as a first language by approximately 70 million people in the country and as a second language by nearly 100 million people across the globe. It is the world’s eleventh most widely spoken language and the most commonly spoken one on the subcontinent [38]. Urdu (native name: اردو) has its roots in the Indo-European language family and is characterized by a distinct morphological and syntactic structure that incorporates elements of Persian, Sanskrit, Arabic, and Turkish, making it challenging to comprehend [39]. In Pakistan, the Supreme Court has mandated that Urdu is recognized as an official language worldwide. Like Persian and Arabic, Urdu is written in the Nastaleeq format from left to right and includes more phonic sounds.

Hindi, a national language of India that is mutually intelligible with the Urdu language, is written in the Devanagari script [40]. Due to Urdu’s complex morphology and limited linguistic resources, the Roman script (i.e., a mashup of two languages using English alphabets) was used to write the content. Urdu, written in Roman script, is known as Roman-Urdu [41], whereas, for the Hindi language, the Romanagari script is used [14].

Urdu is a relatively sophisticated language with a morphological and syntax structure that combines Persian, Sanskrit, English, Turkish, and Arabic [42]. Another prominent feature of the Urdu language is its contextual sensitivity. In Urdu, a letter can be written in more than one shape, with the shape decided by two factors: the position of the letter and the surrounding letters [43].

Little effort was made into Urdu language processing due to a scarcity of resources. A few survey papers on Urdu and associated subjects have been conducted. Riaz et al. [44] explored novel strategies for Urdu language challenges such as Stop Words Recognition, Stemming, Concept Searching, and Named Entity Recognition (NER). Recently, in the Asian language processing research community, researchers have gained much insight into Urdu. Despite having over 100 million speakers worldwide, Urdu is a resource-limited language in the NLP domain, both in terms of NLP tool accessibility and the lack of labelled datasets [45].

Despite the deep learning and ML approaches and the outlined performance they achieved, these approaches are mainly focused on the English language. There are few methods for identifying sarcasm in Urdu, but more research and analysis are needed to improve their reliability.

## 3 Proposed research approach

This study explores tweets from user-generated comments on 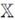, previously Twitter, explicitly concentrating on low-resource languages. We first elaborated on the proposed research questions aimed to be answered through the proposed research methodology. Secondly, we describe the research data and the identified features to describe the research approach.

### 3.1 Research questions

The research aims to address the challenges by investigating the linguistic cues, cultural nuances, and computational methodologies specific to Urdu sarcasm detection. By exploring diverse ML techniques and leveraging linguistic features inherent to low-resource language, this study aims to advance the development of automatic sarcasm detection systems tailored for the low-resource language. Below, we formulated three research questions to better understand the purpose of the research approach.

**RQ1** How do Urdu linguistic nuances and cultural references influence sarcasm detection?

**RQ2** What are Urdu’s unique features or linguistic patterns that aid in identifying sarcastic content?

**RQ3** How do different ML models perform in detecting sarcasm in Urdu?

In a nutshell, RQ1 revolves around an extensive analysis of user comments. It aims to identify the common patterns in end-user tweets generated for low-resource languages, explicitly focusing on Urdu. RQ2 utilizes grounded theory and a content analysis approach to identify diverse features and linguistic patterns commonly used in Urdu to represent sarcasm in low-resource languages. Conversely, RQ3 aims to assess and compare the efficacy of various baseline machine-learning classifiers in automatically detecting sarcasm within low-resource languages. The goal is to scale and automate the proposed approach for sarcasm identification for low-resource languages.

### 3.2 Research method

**Fig 1:**
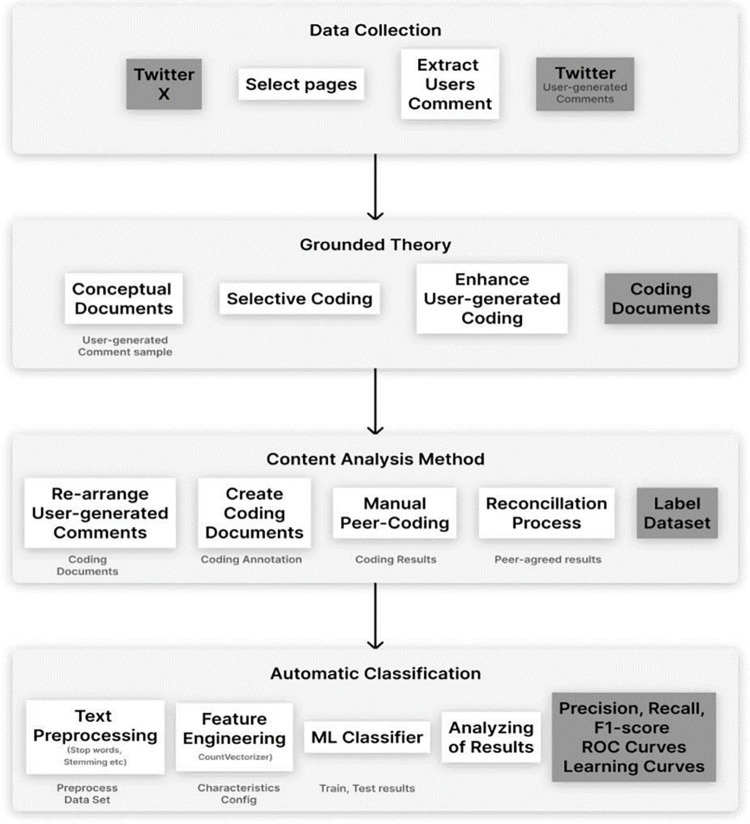
Overview of the proposed research methodology.

The proposed research approach comprises four main phases and goals, as shown in Fig 1; each methodological step is elaborated below.

#### 3.2.1 Data collection and development

Recently, there has been a surge in proposing novel research approaches utilizing low-resource languages, such as Hindi, Arabic, and Urdu, for various purposes, i.e., sentiment analysis, opinion missing, and other NLP-related tasks. Urdu is widely recognized as a low-resource language, as several computational resources are unavailable or inaccessible [46]. In line with that, we are interested in exploring sarcasm identification in the low-resourced Urdu language in the online user-generated data that have been given less concentration in the literature. However, we were confronted with a data availability problem for sarcasm detection. Therefore, we developed a research data set comprising user tweets from 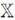 social media platforms publicly available for research purposes. We target the social media platform that people mostly use to express their opinions using their native languages. Also, there is a scarcity of Urdu sarcastic datasets. We curated and released a comparatively large dataset named Urdu Sarcastic Tweets (UST) Dataset, comprising user-generated comments from 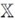. Additionally, we argue that the proposed research data set dataset for sarcasm detection in Urdu is the first publicly available Urdu-sarcastic-tweets-dataset https://www.kaggle.com/datasets/shumailakhan/urdu-sarcastic-tweets-dataset of Urdu sarcastic tweets.

We have collected data by scrapping 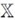 for ’tweets’ and ’replies’ written in Urdu. We utilized a Python-based online library, Tweepy, to crawl Urdu tweets from 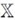. As part of configuring the data retrieving process, we selected the Urdu language. To determine the search query, we iterated through a list of trending hashtags representing various aspects of sports and political tweets. The sources for data collection include BBC Urdu Urdu News, as well as images shared by the Pakistani and Indian public, which were collected from 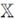. The dataset contains tweet information, including tweet ID, text, gender, emoji/emoticons, date and time, and location. A Python script extracts emojis from tweet text, gathering their descriptions and sentiment scores [47]. The tweets are encoded in UTF-8 (Unicode Transformation Format version 8) and saved in a text file. The research dataset is balanced, comprising several instances under sarcastic and non-sarcastic categories.

#### 3.2.2 Grounded theory approach

In this step of the proposed methodology, a coding guideline document is developed by manually analyzing the test sample of collected tweets from X in the dataset. Grounded theory is a qualitative research methodology aiming to develop theories based on the frequently occurring concepts in data collected systematically from the participants. Also, to develop an automated approach capable of classifying social media text written in a low-resourced Urdu language into sarcastic and non-sarcastic instances, we need to build a grounded theory that can provide a systematic and comprehensive understanding of how individuals can differentiate between sarcastic and non-sarcastic tweets. The coding guidelines document outlines the process for conducting grounded theory research and the coding procedures for analyzing data related to Urdu sarcasm detection. The final output is then used to label the dataset in the content analysis phase, contributing to minimizing the disagreement between the different annotators when labelling the dataset for the ML experiments, as shown in Fig 1. The developed coding guideline includes definitions of each fundamental concept with examples and instructions for annotating user tweets within the dataset. Also, the coding process is conducted iteratively. The first two authors of the paper discuss the coding guidelines, refine them, and resolve disagreements through discussions and reconciliation sessions, if any, enhancing and stabilizing them.

#### 3.2.3 Analyzing content manually

After developing the coding guideline, the third step in the proposed approach is to annotate the user tweets in the dataset using a content analysis approach. For this purpose, the first three authors of the paper manually annotated the user tweets in the dataset to make the data pars able for the ML classifiers. The proposed approach annotation process is inspired by Riloff et al. [48] and the content analysis approach [49]. We aim to answer RQ1 by focusing on Urdu’s linguistic nuances and cultural references, highlighting how sarcasm manifests in specific linguistic contexts, deepening our understanding of language-specific cues, and enriching sentiment analysis. In the exploration to discern language-specific cues within Urdu that distinguish between sarcastic and non-sarcastic comments, RQ2 aligns to identify linguistic features inherent to Urdu. Investigating these nuanced aspects within Urdu allows for a deeper grasp of how sarcasm manifests within language, including cultural references, idiomatic expressions, or linguistic subtleties. However, identifying the most practical features or linguistics patterns for resource-less languages is also challenging due to language diversity, data sparsity, noise, limited annotated data, cultural nuances and context, the complexity of sarcasm, and the lack of computational tools. In addition to addressing RQ3, which involves assessing the effectiveness of ML models in sarcasm detection within Urdu, essential for identifying the most suitable models for this language, our study tackles the intricacies inherent in sarcasm detection within low-resource languages.

During the content analysis phase, several crucial steps were performed: First, we created a sample dataset from the collected dataset. Secondly, an annotation form was created using Microsoft Excel to annotate the human annotators’ user-generated comments. These annotators utilized the developing code guidelines and identified features for the annotation process. Thirdly, the selected human coders had the annotation form and coding guidelines to annotate each comment. We used inter-annotator agreement to measure the level of agreement between three human annotators who independently annotated the same data set. It is often used in NLP and ML tasks where data needs to be labelled with predefined categories or annotations. The inter-annotator agreement can be measured using various metrics such as Cohen’s kappa, Fleiss’ kappa, and Krippendorff’s alpha [50].

#### 3.2.4 Automated classification

The final phase of the proposed research approach aims to identify how well we automatically classify sarcasm in Urdu. For this purpose, we executed the subsequent steps. Initially, we conducted the pre-processing steps, including stop words removal, punctuation marks, unnecessary characters, hashtags, URLs, digits, all non-Urdu words, and special characters using iNLTK https://inltk.readthedocs.io/en/latest/and NLP Toolkit https://www.nltk.org/. After the pre-processing, we employed feature engineering techniques to convert textual data into numerical format to understand ML models. The pseudocode for the methodology process is depicted in Algorithm 1. To accomplish this task, we implemented TF-IDF and CountVectorizer techniques on the textual data. Finally, to evaluate the performance of ML techniques in automatically classifying sarcasm in text, we used standard evaluating matrices like receiver operating characteristic curve (ROC), confusion matrix, precision, recall, and a harmonic mean known as the F1-Score.

Algorithm 1: Data Pre-processing and Processing Algorithm

**Step 1**: Collect Data

data = CollectDataFromXPlatform()

**Step 2**: Identify Data Sources

sources = IdentifyDataSources()

**Step 3**: Ensure Data Availability

dataAvailabilityIssues = RecognizeDataAvailabilityIssues()

**Step 4**: Manually Annotate Dataset

codingGuidelines = DevelopCodingGuidelines()

annotatedDataset = AnnotateDatasetManually(data, codingGuidelines)

**Step 5**: Manual Analysis of Dataset Content

analysisResults = AnalyzeContentManually(annotatedDataset, codingGuidelines)

**Step 6**: Pre-process Text Data

nlpLibrary = "SpaCy"

preprocessingSteps = ["Tokenization", "Stopword Removal", "Lowercasing"]

**for** each tweet in annotatedDataset:

       # Apply specified pre-processing steps using NLP library

       preprocessedTweet = PreprocessText(tweet, nlpLibrary, preprocessingSteps)

       preprocessedDataset.append(preprocessedTweet)

**Step 7**: Extract Textual Features

featureExtractionTechniques = ["Countvectorization"]

**for** each tweet in preprocessedDataset:

       # Extract specified textual features using chosen techniques

       extractedFeatures = ExtractFeatures(tweet, featureExtractionTechniques)

       featureExtractedDataset.append(extractedFeatures)

**Step 8**: Handle Emojis

emojiLibrary = "Emoji"

transAPI = "Google Translate"

**for** each tweet in featureExtractedDataset:

       # Detect and translate emojis for better understanding

       translatedTweet = HandleEmojis(tweet, emojiLibrary, transAPI)

       datasetWithTranslatedEmojis.append(translatedTweet)

**Step 9**: Handle Slangs

urbanDictionary = "UrbanAPI"

**for** each tweet in datasetWithTranslatedEmojis:

       # Replace slangs and abbreviated words with their original forms

       originalSlangsTweet = HandleSlangs(tweet, urbanDictionary)

       datasetWithOriginalSlangs.append(originalSlangsTweet)

**Step 10**: Augment Data

augmentationTechniques = ["Synonym Replacement", "Back Translation"]

augmentedDataset = []

**for** each technique in augmentationTechniques:

       **for** each tweet in datasetWithOriginalSlangs:

              # Apply specified data augmentation technique

              augmentedTweet = ApplyAugmentation(tweet, technique)

              augmentedDataset.append(augmentedTweet)

**Step 11**: Save Pre-processed Data to File

SaveDatasetToFile(augmentedDataset, "UTF-8", "preprocessed_data.txt")

## 4. Dataset processing

In this section, our focus lies on the labelling mechanism employed for user-generated tweets. The goal is to detect and capture sarcastic elements within user-generated tweets from 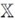, particularly those associated with low-resource language.

### 4.1 Users statements

In the 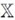 dataset, users’ tweets can be classified into various concepts by analyzing them based on their behavior exhibited on social media. Specifically, for the proposed approach, our interest lies in extracting tweets that demonstrate diverse user behaviour, contextual understanding, diversity, and sarcasm in Urdu. This step involved a comprehensive analysis and evaluation of user-generated comments to identify how users submit sarcastic comments in low-resource language. Also, a unique annotation coding guideline has been developed, which will be utilized by potential coders when annotating the tweets for the machine learning classifiers. The coding guidelines document outlines the concepts, including annotation codes derived from frequently used phrases ("بہت ہی عمدہ", "واہ واہ"), words with irony ("اچھا", "تعریف"), and special characters, all chosen for their relevance to the research objectives. Each of the recognized annotation codes is described below.

#### Sarcastic sentences

The code of "sarcastic" is assigned to user tweets written in Urdu that portray an opposite meaning to what they are saying, having the intention to insult or mock someone. Sarcasm often conflicts between explicit expressions and the intended meaning, relying on subtle cues, such as tone or context, to convey the underlying irony. For example, "بہت ہی عمدہ کام کیا آپ نے!!!!!!!", and “واہ! کیا خوبصورتی ہے آپ کی!", both apparently praising the subject. However, the exaggerated usage of exclamation marks in the first sentence and the seemingly enthusiastic tone in the second sentence indicate sarcasm. These linguistic elements contrast the intended meaning and the literal interpretation. Sarcasm in Urdu exploits these linguistic inconsistencies to convey contradictory yet nuanced sentiments, adding depth and layers to the communicated message. Understanding these nuances is essential to incorporate sarcastic expressions within Urdu.

#### Non-sarcastic sentences

The code of non-sarcastic is assigned to user tweets that demonstrate straightforward meaning, i.e., are easy to interpret and understand by reading them and show the same meaning the user wants to portray. In contrast to sarcastic expressions, non-sarcastic sentences do not require a deeper interpretation of linguistic cues. They present clear, unambiguous information, making them easily understandable. Understanding the distinction between non-sarcastic and sarcastic expressions in Urdu is essential for effective communication and accurate interpretation of intended meanings within conversations and written text. For example, “ہمیشہ اچھے کام کرو", "میں نے کتاب پڑھی". These sentences convey straightforward and literal meanings without any ironic or sarcastic intent. They express typical statements or factual information in Urdu. Detecting sarcasm in Urdu often relies on recognizing discrepancies between the explicit words used and the speaker’s underlying tone, context, or intent.

#### Annotation criteria

The following criteria are identified when annotating the user tweets into sarcastic and non-sarcastic.

- Sarcasm means using language to convey the opposite of the literal meaning, often mocking, criticizing, or expressing irritation. For example,

" ہاں، حکومت نے تو ہمیشہ کی طرح چمکتے ہوئے منصوبے شروع کیے ہیں، جو ہمیشہ کی طرح مکمل ہوتے ہیں"۔

- Tone and Context: Detecting Urdu sarcasm requires comprehending contextual intricacies, lexical variations, and intricate linguistic nuances. i.e.

اس شخص کو دیکھو، ہر بار وعدے کرتا ہے کہ دنیا بھر کو بہتر بنائے گا۔""

"ہاں بلکہ ہمیں تو اب تک کچھ نظر نہیں آیا اس کی خدمات سے!"

- Linguistic Devices: The annotators are also guided to annotate tweets as sarcastic when a negative situation follows a positive sentiment or when contempt emojis follow positive tweets and vice versa, as these are considered indicators of sarcasm. Identify linguistic cues such as exaggerated punctuation (e.g., excessive exclamation/question marks), unexpected word usage, or contradictory expressions that signify sarcasm. Another rule is that Tweets with admiration or exclamation words (like Cheers, Yay) followed by unpleasant emoticons are also considered sarcastic. For example,
  ○ Exaggerated Punctuation:
    "واہ! آپکی حکومت نے کام کرنے کا طریقہ بلکل بے مثال ہے!!!"
  ○ Contradictory Expressions:
    "میرے پیارے نظریے دوست، یہ ٹریفک واقعی مزیدار ہوتا جا رہا ہے۔ #TrafficFun"
  ○ Admiration Words Followed by Unpleasant Emoticons:
    "واہ کیا خوبصورت مناظر ہے! 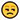
"

These examples describe how linguistic devices like exaggerated punctuation, contradictory expressions, and the combination of admiration words with unpleasant emoticons can be used to convey sarcasm in Urdu tweets.

#### Annotation instruction

All the individuals involved in the annotation process are experienced professors and postgraduate computer science students with diverse data annotation experience. The coders were instructed to disregard digits, symbols, and code-mixed Roman and Arabic texts and solely concentrate on pure Urdu. Furthermore, we specified the required storage format, specifically the UTF encoding. The dataset was labeled by assigning a ’sarcastic’ label for sarcastic tweets and a ’non-sarcastic’ label for non-sarcastic tweets, respectively. The annotated outputs from the collected tweets are used as the "ground truth" to evaluate how effectively the various machine learning algorithms perform in automatically identifying sarcastic tweets in low-resourced language [48].

### 4.2 Challenges in manual annotation of Urdu

Annotating or labeling Urdu for sarcasm detection presents substantial hurdles owing to the language’s diverse linguistic intricacies, cultural nuances, and intricate script complexities. Annotators frequently face difficulties in unraveling the intricate layers of sarcasm or encapsulating the wide array of sentiments expressed in Urdu. Understanding and addressing these challenges in annotating Urdu are pivotal for advancing NLP applications.

#### Subjectivity and interpretation

Annotators’ diverse perspectives lead to varying interpretations of text, resulting in differing annotations for sentiment, tone, or sarcasm. To mitigate this, we have developed a comprehensive coding guideline for annotators. This guideline includes definitions and examples, aiding annotators in navigating the annotation process consistently.

#### Cultural nuances

The presence of intricate cultural subtleties and regional variations within Urdu makes accurate text labeling challenging for annotators unfamiliar with these nuances. We set some basic examples for better understanding.

#### Sarcasm and irony

The identification of sarcasm or irony in Urdu proves intricate due to indirect expressions, diverse cultural backgrounds, and varying degrees of subtlety in conveying sarcasm. The coding guideline offers specific examples and guidelines on identifying indirect expressions, ensuring a more nuanced approach to sarcasm detection.

#### Linguistic complexity

The Urdu script’s intricate calligraphy, diacritics, and script variations pose challenges for annotators and annotation tools alike. Our coding guideline includes visual aids and explanations to help annotators navigate through linguistic complexities, ensuring consistency in annotation.

#### Annotation consistency

Ensuring uniform annotations across multiple annotators becomes daunting due to divergent interpretations and understandings of Urdu language subtleties. The coding guideline acts as a reference point, fostering consistency by providing a shared understanding of annotation principles.

#### Limited labeled data

The scarcity of pre-labeled datasets in Urdu restricts the creation of robust training sets essential for machine learning models. To overcome this challenge, we actively collaborate with language experts and continuously update our coding guidelines to include diverse examples from contemporary sources.

#### Resource intensiveness

Manual annotation necessitates a substantial allocation of human resources, time, and expertise. The coding guideline streamlines the annotation process, maximizing efficiency and ensuring that resources are utilized effectively.

### 4.3 Disagreement and negotiation between the coders

A coding guideline was developed to resolve the disagreement between the coders on annotating the user tweets into sarcastic and non-sarcastic. Also, we used Inter-annotator agreement to measure the level of agreement between three human annotators who independently annotate the same data set. It is often used in NLP and ML tasks where data needs to be labeled with predefined categories or annotations. The inter-annotator agreement can be measured using various metrics such as Cohen’s kappa, Fleiss’ kappa, and Krippendorff’s alpha [50].

Cohen’s kappa is used to measure inter-annotator agreement between two annotators. It is calculated using the following equation:

$$K=\frac{p_{o}-p_{e}}{1-p_{e}}$$ (1)

Where: K is Cohen’s kappa

- *p*_*o*_ is the observed agreement between the two annotators (the proportion of times they agreed).
- *p*_*e*_ is the expected agreement by chance.

To calculate p_o Count the number of times the two annotators agreed on the same label and divide it by the total number of annotations. To calculate p_e, we need to calculate the expected agreement by chance, which depends on the number of categories and the distribution of the categories in the data.

During phase I, a specific subset of data is allocated to a pair of annotators chosen randomly without prior knowledge of the content. These annotators then assign labels to indicate the presence or absence of sarcasm in the tweet being examined. After completing the process, we performed a comparative analysis and computed Cohen’s kappa statistic (k) specifically for the subset related to sarcasm annotation.

In instances where the agreement was lower (0.6), we initiated a repetition of the labeling process for that specific subset. In contrast, if the agreement exceeded 0.6 (k > 0.6), we retained the tweets in the labeled pool where consensus was achieved. In the initial phase of the labeling process, some tweets pose a challenge in determining whether they contain sarcasm. This challenge often arises from the intricate and sometimes subjective nature of sarcasm interpretation. When an agreement cannot be reached between the two annotators assigned to a specific tweet, these tweets are kept aside for phase II to be labeled by the third annotator. A third annotator labeled these tweets in phase II and subsequently included them in the labeled pool based on the majority consensus. The statistics are shown in Table 2.

**Table 2:** Inner annotator agreement distribution.

Metric	Value	Interpretation Agreement
Observed Agreement	0.5	50%
Expected agreement	0.25	225%
Kappa value	0.66	The annotators agree on the labels for the tweets more than would be expected by chance.

## 5. Automated classification of sarcasm in low-resource language

Recently, there has been a surge in the utilization of social media platforms, positively impacting people’s social lives. For example, to get instant information about various events on "
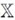
" social media platform, which millions of users use daily. Algorithm 2 depicts the classification process. Also, getting insightful information about user experiences and recommendations about different products and software applications on social media platforms [51, 52], such as product forums [53], app stores [54], and discussion forums [55]. However, despite these advantages, there are numerous challenges in dealing with social media textual data, such as fake reviews that lead to wrong purchase decisions, resulting in the waste of resources and money [52, 53]. Also, users submit sarcastic reviews on these social media platforms that change the intended meaning of the information, which is referred to as deceiving users.

Algorithm 2: Methodology Algorithm

**Step 1**: Define Study Objective

studyObjective = DefineStudyObjective()

**Step 2**: Identify Low-Resource Languages

lowResourceLanguages = IdentifyLowResourceLanguages()

**Step 3**: Develop Coding Guidelines

codingGuidelines = DevelopCodingGuidelines()

**Step 4**: Train and Validate Classifiers

classifiers = ["Random Forest", "Support Vector Machine"]

validationMethod = "Stratified Cross-Validation"

evaluationMetrics = ["Accuracy", "Precision", "Recall", "F1 Score"]

evaluationResults = []

**for** each classifier in classifiers:

       **Step 4.1**: Split dataset into training and validation sets

       trainingSet, validationSet = SplitDataset(augmentedDataset, validationMethod)

       **Step 4.2**: Train classifier on training set

       trainedClassifier = TrainClassifier(classifier, trainingSet)

       **Step 4.3**: Validate classifier on validation set

       validationResults = ValidateClassifier(trainedClassifier, validationSet)

       **Step 4.4**: Evaluate classifier performance using specified metrics

       evaluation = EvaluateClassifier(validationResults, evaluationMetrics)

       evaluationResults.append(evaluation)

**Step 5**: Analyze Results

# Placeholder for further analysis and interpretation of evaluationResults

analysisSummary = AnalyzeResults(evaluationResults)

**Step 6**: Generate Reports

report = GenerateReport(analysisSummary)

**Step 7**: Conclusions and Recommendations

conclusions = SummarizeConclusions(analysisSummary)

recommendations = ProvideRecommendations(analysisSummary)

**Eq 1**: Overall Study Performance Metric

overallMetric = CalculateOverallMetric(evaluationResults)

$Overall_{Metric}=\frac{sum(evaluationResults)}{len(evaluationResults)}$

**Eq 2**: Performance Comparison

performanceComparison = ComparePerformance(classifiers, evaluationResults)

$Performancce_{Comparision}=evaluationResults[0]-evaluationResults[1]$

The large number of feedback submissions to these platforms makes it challenging to process and identify fake and sarcastic information manually. The situation worsens for low-resource languages in these social media platforms due to a lack of researchers’ interests and resources such as datasets. Therefore, to counter these challenges, we processed user tweets on the "
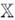
" platform and identified that many users submit sarcastic reviews on certain categories, such as politics and support. To process such a large number of feedback, we are interested in employing various classification algorithms to identify their performance in automatically identifying sarcastic reviews written in low-resource languages, such as Urdu. For this purpose, we developed an annotated UST dataset. The ML models included support vector machine (SVM) with different Kernel types, decision tree (DT), K-Nearest Neighbor Classifier (K-NN), linear regression (LR), random forest (RF), Naïve Bayes (NB), and XGBoost. Instead of systematically evaluating all the possible configurations according to the relevant research, one of the main objectives was to find configurations that result in accurate classifiers for identifying sarcasm in Urdu. This article aims to analyze and appraise the accuracy of different ML techniques. However, we achieved a relatively high precision, recall, and F1-score when deploying different ML models with different configurations. The details of the ML experiments are discussed below.

### 5.1 Experimental setup

This section describes various tools and configurations used to implement the proposed ML model evaluation to identify sarcasm in Urdu. The experiments were conducted in a Colab Pro environment featuring Intel Core i7-9700M, 3.6GHz as shown in Table 3. All the techniques were employed using Python 3.8, Scikit-learn, Numpy and NLTK.

**Table 3:** System configuration.

Hardware and Software	Specifications
Processor	Intel Core i7-9700k, 3.6GHz
GPU	GeForce RTX 2080 Ti, 11GB
RAM	32GB DDR4
Operation System	Windows 11 x64
Environment	Python: 3.8, Scikit-learn 0.24.1, XGBoost 1.3.2, Pandas 1.2.3, Numpy 1.20.1, NLTK 3.5
Storage	1 TB SSD

A comprehensive review of automatic sarcasm detection in low-resource language reveals the prevalence of certain classification algorithms, such as NB, SVM, LR, and RF, perform better in processing short text from social media platforms [49, 50]. These techniques, along with additional features like linguistic, behavioral contradiction, and lexical aspects, exhibit superior performance in sarcasm detection [13, 48, 56]. Therefore, before proceeding to text pre-processing, we first select the same ML algorithms for low-resource languages such as roman Urdu based on their performance in text analysis for the English language corpus. The selected algorithms are support vector machine (SVM), decision tree (DT), K-Nearest Neighbor Classifier (K-NN), linear regression (LR), random forest (RF), Naïve Bayes (NB), and XGBoost. The findings indicated comparable accuracy levels among most algorithms. These experiments were executed within a Python environment. Below, we performed a series of steps to prepare the low-resource language data parable for the ML classifiers.

In this study, we utilized a variety of tools and configurations to implement ML models for identifying sarcasm detection in Urdu. the following system specifications are included:

#### 5.1.1 Text pre-processing

Cleaning input data (textual data) is considered significant for the ML experiments. For this purpose, we performed a series of steps as discussed below.

- *Data cleaning*. For cleaning Urdu, various functions can be applied to enhance the quality and consistency of the data. i.e. to remove special characters and eliminate HTML symbols, URLs, and digits, the re (regular expression) module, NLTK, and spaCy are used, which is part of the Python standard library.
  In Urdu, the conversion of lowercase does not result in any visible changes, as Urdu does not have distinct uppercase and lowercase forms for most of its characters. However, this operation is still performed for consistency, normalization, and alignment with common practices in text processing, where case consistency is often desirable. The "lower()" function is a built-in method in Python that converts all characters in a string to lowercase. This operation is language-agnostic and works for Urdu as well.
- *Stop words*. Stop words are frequently occurring words in a language with no semantic value. We used the list of stop words created by Humayoun et al. [57]. Examples of frequently used stop words are listed in Table 4.
- *Tokenization*. Tokenization is breaking down text data into individual words or tokens, which can be utilized as features in ML algorithms. This technique aids in reducing the dimensionality of the text data. Tokenization helps to improve the classification models’ accuracy by capturing the essential linguistic features of the text data. To prepare the dataset for ML processing, the iNLTK python library performed various NLP tasks such as vector embedding, tokenization, and sentence similarity. The library provides the functionalities a developer would require to build NLP applications. Since iNLTK supports Urdu, it was a suitable choice for processing the Urdu sarcastic tweets dataset.

**Table 4:** List of Urdu stop words.

Urdu	Roman-Urdu	English
اگر	Agar	If
آئے	Aay	Came
آج	Aaj	Today
آدهی	Aadhi	Half
آًب	Aab	Now
اکثر	Aksar	Often
آٹھ	Aath	Eight
اور	Aur	And
تر	Ter	-
تو	Tu	so
توبم	Tu ham	So we
ب	Ba	B
اچھی	Archive	Good
اکیلی	Aakeli	Alone
اگرچہ	Agarcha	Although
آخر	Akhir	Finally
آخرکبر	Akhir kab	After all

#### 5.1.2 Feature extraction

In the proposed methodology, we extract the most frequent textual features that perform well on short text-like tweets [58]. The most common textual features including Bag of Words (BoW), Term frequency- Inverse Document Frequency (TF-IDF), and N-grams with ML classifiers [51].

The BoW approach involves creating a dictionary including all words within the corpus. Each document is represented by a vector, where each element corresponds to the frequency of a specific word in the document. Similarly, the TF-IDF technique operates on the premise that words occurring frequently in the text. It considering both the frequency of a word in a document (term frequency) and its inverse document frequency (which reduces the weight of common words). The TF-IDF value increases with the number of times a word appears in the document but decreases with the word’s frequency in the entire corpus.

N-grams are continuous sequence of n-words from a given text. Essentially, they help capture the context by considering word combinations like uni-grams (n = 1): "Cherries" "are", "red". Where bi-grams (n = 2): "Cherries are", "are red", and tri-grams (n = 3): "Cherries are red". By incorporating N-grams as features enhance model accuracy by providing more context compared to single words in detecting sarcasm in Urdu [14].

For the number of n continuous values, n-gram can be calculated as

Ngrams=X−(N−1) (2)

Where x represents occurrences of words in a sentence

N = the number of continuous words.

- *Count vectorization*. A technique used in natural language processing (NLP) for converting a collection of text documents into numerical vectors. Each document is represented as a sparse vector of word counts. Each vector element corresponds to a particular word in the corpus, and the value represents the frequency of that word in the document. For example: "Cherries are red,” can be represented as [1,0,1]
- *Emoji handling*. In the context of sarcasm detection, emojis play a significant role by providing nuanced information that is often essential for understanding the true sentiment. A Python Online emoji library https://github.com/github/gemoji, Google Trans API, and emoji 0.6.0 were used to detect and translate emojis into text. By translating the emojis into text, the model can better understand the sentiment of the text and make more accurate predictions. For example: Λ might be translated to "sad.” This process not only enhances the fluency and clarity of the text but also ensures that ML algorithms can effectively process and analyze the nuanced sentiments conveyed through emojis.
- *Slangs/short abbreviated words*. Slangs and abbreviated words are informal and non-standard words or phrases commonly used in social media and text messages and can be challenging for NLP models. We utilize Urban Dictionary https://www.urbandictionary.com/, replacing short/abbreviated words with their root/original form, enhancing the proposed system’s efficiency and accuracy. For example, the slang "IMO" might be replaced with "in my opinion".
- *Data augmentation*. To increase the diversity of the dataset to get accurate performance, various data augmentation techniques were applied using NLTK and the transformer https://huggingface.co/ library:
  - Back translation, wherein the original Urdu was translated into English and then back into Urdu, ensuring the augmented data’s uniqueness, introducing variations while maintaining the original meaning.
  - Synonym replacement was utilized to randomly replace words with their synonyms.
  - Random deletion selectively eliminated words based on a predefined threshold,
  - Random swaps involved shuffling the order of words.

#### 5.1.3 Assessment & training

We used a stratified fivefold cross-validation approach to the textual data to train and validate the supervised ML algorithms. Four folds of the cross-validation approach were employed to train the ML algorithms, and one fold of cross-validation was used to validate the algorithm. The benefit of the cross-validation approach for training and validating an ML classifier is to check how well a model works if limited data is available. Currently, the stratified K-fold cross-validation approach is most commonly and frequently used to train and validate ML classifiers. Each fold has roughly the same proportion of labels representing each class. To assess the effectiveness of the classifiers, we compute and summarize the average results obtained from the tenfold cross-validation runs.

The classifier accuracy can be measured by evaluating different performance parameters. The most common parameters for text classifications are accuracy, F-measure, precisions, recall, and AUC.

*Accuracy (ACC)*. ACC shows the percentage ratio of the predicted instance. It measures overall correctly classified instances. Mathematically, accuracy can be described as:

$$Acc=\frac{TP+TN}{TP+TN+FP+FN}$$

Where;

TP: True Positive, and is predicted positive.

TN: True Negative and is predicted negative.

FP: False Positive predicted positive but false actually.

FN: False Negative predictions are false but true.

*Precision (Pre)*. The computation ratio of true positive over the positive result.


$$Pre=\frac{TP}{TP+FP}$$

*Recall (Rec)*. The recall is the percentage of actual positives that are expected to be positive. It represents the ratio of true positives to all true.


$$Rec=\frac{TP}{TP+FN}$$

*F-measure (F1)*. The F1-measure represents the harmonic mean of precision and recalls when false positives and false negatives are equal. The standard F-measure values precision and recall equally.


$$F1\;measure=2\times \frac{Pre\times Rec}{Pre+Rec}$$

#### 5.1.4 Experimental results

This section unveils groundbreaking findings that underscore the efficacy of the proposed machine-learning approach for sarcasm detection in Urdu. Through rigorous evaluation across diverse classifiers and feature engineering techniques, the study establishes Linear Regression and Random Forest as superior performers, achieving remarkable accuracy of 0.89 and 0.88, respectively. This breakthrough not only validates the robustness of the methodology but also paves the way for future advancements in sentiment analysis and natural language processing for low-resource languages like Urdu. To comprehensively explore sarcasm detection in Urdu tweets, we employed two different experiments, i.e., a featured-based N-gram approach and a pipeline architecture using textual features. Each setup serves a unique purpose and contributes complementary insights into the intricacies of sarcasm detection.

The need for a comprehensive exploration of sarcasm detection methodologies determines the choice of two different experiments. The feature-based approach allows a deep dive into the impact of specific linguistic structures (N-grams) on classifier performance. On the other hand, the pipeline architecture provides a broader assessment of the overall effectiveness of diverse machine-learning algorithms. Together, these setups offer a well-rounded understanding of sarcasm detection in Urdu tweets—from the intricacies of language structures to the holistic evaluation of classifiers in a unified framework.

- *Featured-based approach with N-grams*. The proposed featured-based approach incorporates n-grams, a contiguous sequence of ’n’ items from a given text sample, for effective feature extraction in Urdu, as shown in Table 5. Specifically, we applied SVM, Multinomial NB, Bernoulli NB, XGBoost, DT, RF, and K-NN, tailoring each to different n-gram types. Surprisingly, the performance of the proposed approach when using uni-gram (n = 1) features on the proposed dataset, we found that most of these classifiers have effective results in performance metrics. Interestingly, SVM-Countvectorization-unigram and Bernoulli NB-Countvectorization-unigram perform best, achieving high accuracies of 0.81 and 0.80, respectively. These results show that, for the uni-gram feature extraction method, SVM and Bernoulli NB are the most effective classifiers for sarcasm detection in Urdu.

**Table 5:** Results of featured-based classifiers.

Classifier	Classifier Features	Precision	Recall	F1-score	Accuracy
SVM	Countvectorization-ngram	**0.80**	**0.89**	**0.84**	**0.81**
	Countvectorization-Bigram	0.79	**0.90**	0.75	**0.81**
	Countvectorization-trigram	0.77	**0.91**	**0.83**	0.79
DT	Countvectorization-ngram	0.70	0.63	0.66	0.63
	Countvectorization-Bigram	0.69	0.72	0.71	0.66
	Countvectorization-trigram	0.63	**0.88**	0.76	0.69
K-NN	Countvectorization-ngram	**0.95**	0.20	0.33	0.54
	Countvectorization-Bigram	**0.96**	0.14	0.24	0.51
	Countvectorization-trigram	0.95	0.14	0.24	0.51
RF	Countvectorization-ngram	**0.86**	**0.90**	0.87	0.77
	Countvectorization-Bigram	0.57	1.00	0.73	0.57
	Countvectorization-trigram	**0.86**	**0.90**	**0.87**	**0.77**
LR	Countvectorization-ngram	0.77	0.91	0.83	0.79
	Countvectorization-Bigram	0.74	0.92	0.82	0.77
	Countvectorization-trigram	0.74	0.92	0.82	0.77
Multinomial NB	Countvectorization-ngram	0.79	0.79	0.79	0.76
	Countvectorization-Bigram	0.77	0.87	0.82	**0.78**
	Countvectorization-trigram	0.74	**0.92**	0.82	**0.77**
Bernauli NB	Countvectorization-ngram	0.79	**0.88**	**0.83**	**0.80**
	Countvectorization-Bigram	0.74	**0.95**	0.83	0.78
	Countvectorization-trigram	0.70	**0.98**	0.82	0.75
XGBoost	Countvectorization-ngram	0.79	**0.84**	**0.82**	0.78
	Countvectorization-Bigram	0.79	0.84	0.82	**0.79**
	Countvectorization-trigram	0.78	**0.85**	**0.82**	0.78

SVM-Countervectorizer-bigram performed well with an accuracy of 0.81. Preferably, the K-NN-Countervectorize-bigram achieved the highest precision at 0.96, though its overall accuracy remains lower at 0.51. MNB, BNB, and XGBoost remain consistent at an accuracy of 0.78 or 0.79. In comparison, the DT and RF got moderate accuracy. Furthermore, for tri-gram, the SVM classifier has the highest accuracy of 0.79 among all the classifiers. While K-NN stands out with the highest precision of 0.95, its accuracy is 0.50, indicating many false positives. RF has a good recall of 1.00, representing that the positive samples were identified correctly. However, its precision and F1-score are relatively lower. Meanwhile, multinomial NB, Bernoulli NB, and XGBoost remain slightly consistent with an accuracy of 0.78 or 0.77.

**Fig 2:**
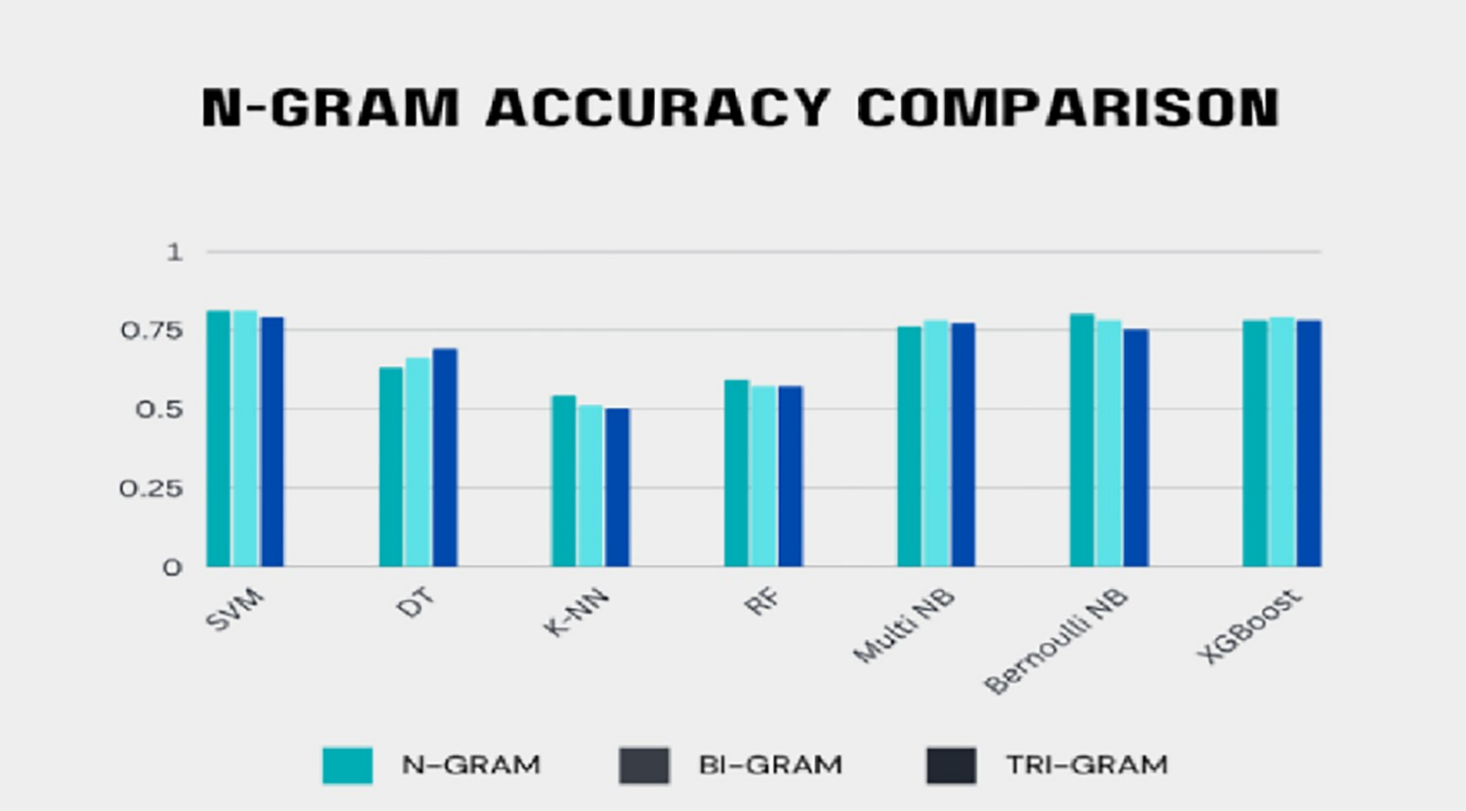
Comparison of n-gram models.

Uni-gram accuracy is better than bi-gram and tri-gram because a single word is most prominent in the text. Fig 2 depicts the performance of the suggested model’s n-gram feature. This graph depicted only the accuracy of using uni-grams, bi-grams, and tri-grams with various ML classifiers. The proposed n-gram feature evaluation results suggest that the value at n = 1 is superior to that at n = 2 and n = 3. We can determine the true meaning of the sentence if it is completed (which means both the subject and predicate are present in the sentence); otherwise, the sentence is regarded as ambiguous. So, the entire sentence is considered to analyze the optimum results for sarcasm detection. It is challenging to examine the effects of a single word when considering the n-gram because the word’s impact on the sentence depends on whether it is positive or negative.

*(B) Pipeline architecture using textual features*. In this phase, we adopted a pipeline approach to encapsulate the whole process of sarcasm classification. Multiple ML classifiers, including SVM, RF, LR, Bernoulli NB, Multinomial NB, and DT, are integrated into a unified workflow. The pipeline approach enables a comprehensive comparison of different classifiers, considering their overall performance in the sarcasm detection task. The results obtained from various machine learning algorithms in the sarcasm classification experiments are presented in Table 6, with bold values indicating the highest scores for each evaluation metric. LR and RF algorithms demonstrated relatively high accuracy, achieving 0.89 and 0.88, respectively.

**Table 6:** Results analysis of ML textual classifiers.

Classifier	Precision	Recall	F1-score	Accuracy
SVM	0.78	0.79	0.78	0.79
DT	0.78	0.75	0.76	0.76
K-NN	0.95	0.10	0.17	0.71
RF	**0.84**	**0.85**	**0.85**	**0.85**
Multinomial NB	0.70	0.79	0.79	0.78
Bernaulli NB	0.78	0.82	0.80	0.79
LR	**0.82**	**0.82**	**0.82**	**0.82**
XGBoost	0.85	0.78	0.82	0.78

**Fig 3:**
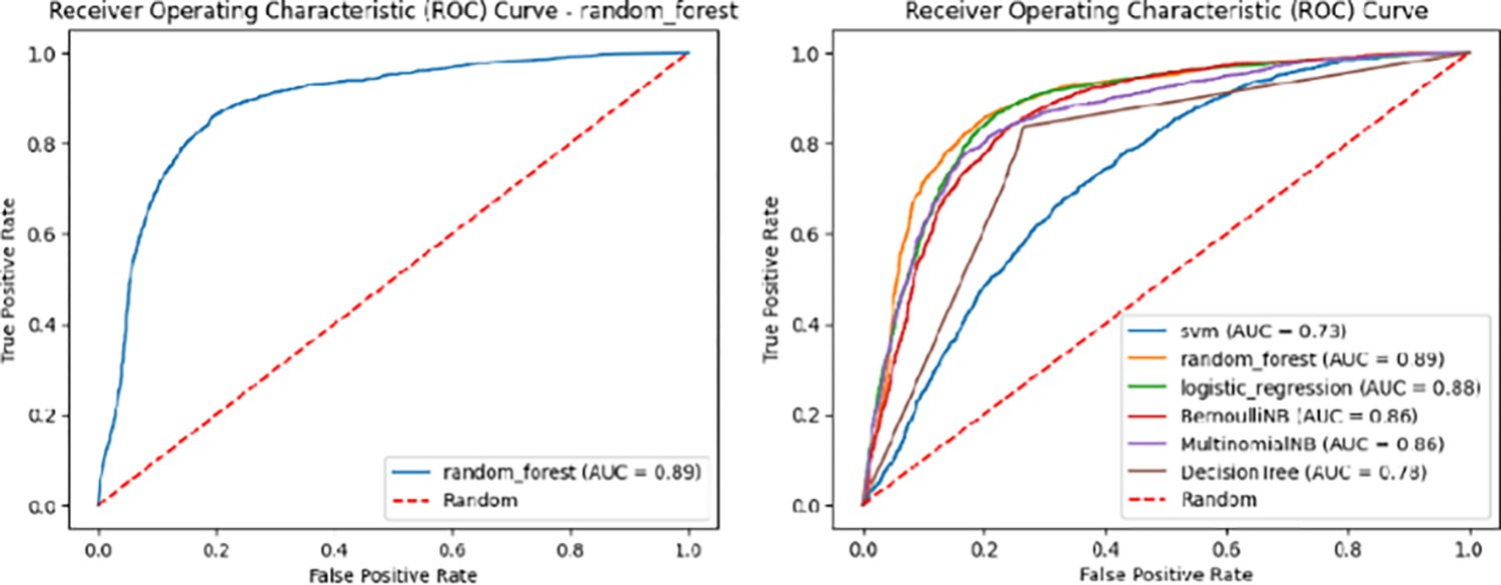
ROC curve showing the performance of ML classifiers.

The ROC curves for LR and RF are shown in Fig 3. The ROC curve is a plot of the true positive rate (TPR) versus the false positive rate (FPR) of a classification model at different classification thresholds. The TPR is the proportion of positive cases that the model correctly classifies, and the FPR is the proportion of negative cases that are incorrectly classified as positive by the model. An ideal ROC curve would be a diagonal line from the bottom left corner of the graph to the top right corner. This would indicate that the model can distinguish between positive and negative cases perfectly.

**Fig 4:**
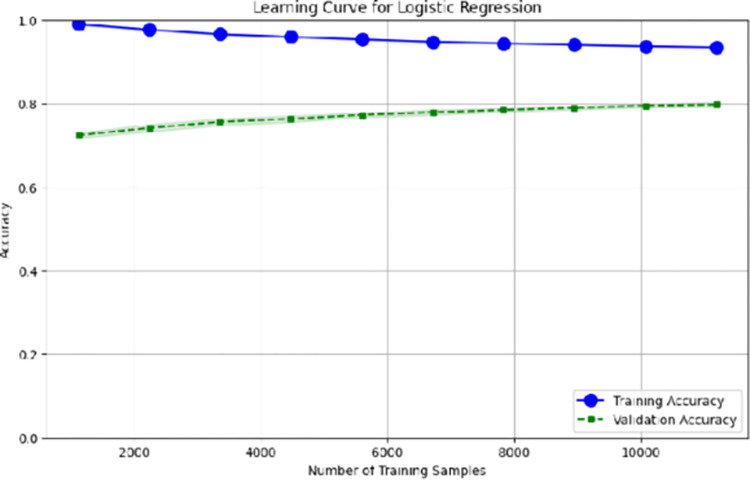
Learning curves to train the machine learning classifier.

Both Multinomial NB and Bernoulli NB also performed well, reaching an accuracy of 85%. Although SVM showed relatively high performance when utilizing feature n-grams, its results with textual features were unsatisfactory, exhibiting lower accuracy compared to other algorithms. The figures suggest that SVM requires more generalization. The training and validation curves for RF and LR are shown in Fig 4. The learning curve shows that the training accuracy and validation accuracy both increase as the number of training samples increases. This is because the model can learn more about the features that are important for classifying sarcasm as the number of training samples increases. The training accuracy is always higher than the validation accuracy.

For Urdu classification, LR and RF stand out as the best-performing algorithms. When classifying user-generated comments into sarcastic and non-sarcastic categories, LR consistently outperforms other machine learning classifiers. However, the choice of algorithm depends on the application’s specific needs, such as whether it is more important to minimize false positives or false negatives. It is also worth noting that the performance of these algorithms may be enhanced further by fine-tuning the hyperparameters with approaches like grid search or random search. In addition, with the performance metrics, learning curves were generated to provide additional insights into the training and validation performance of the proposed model.

**Fig 5:**
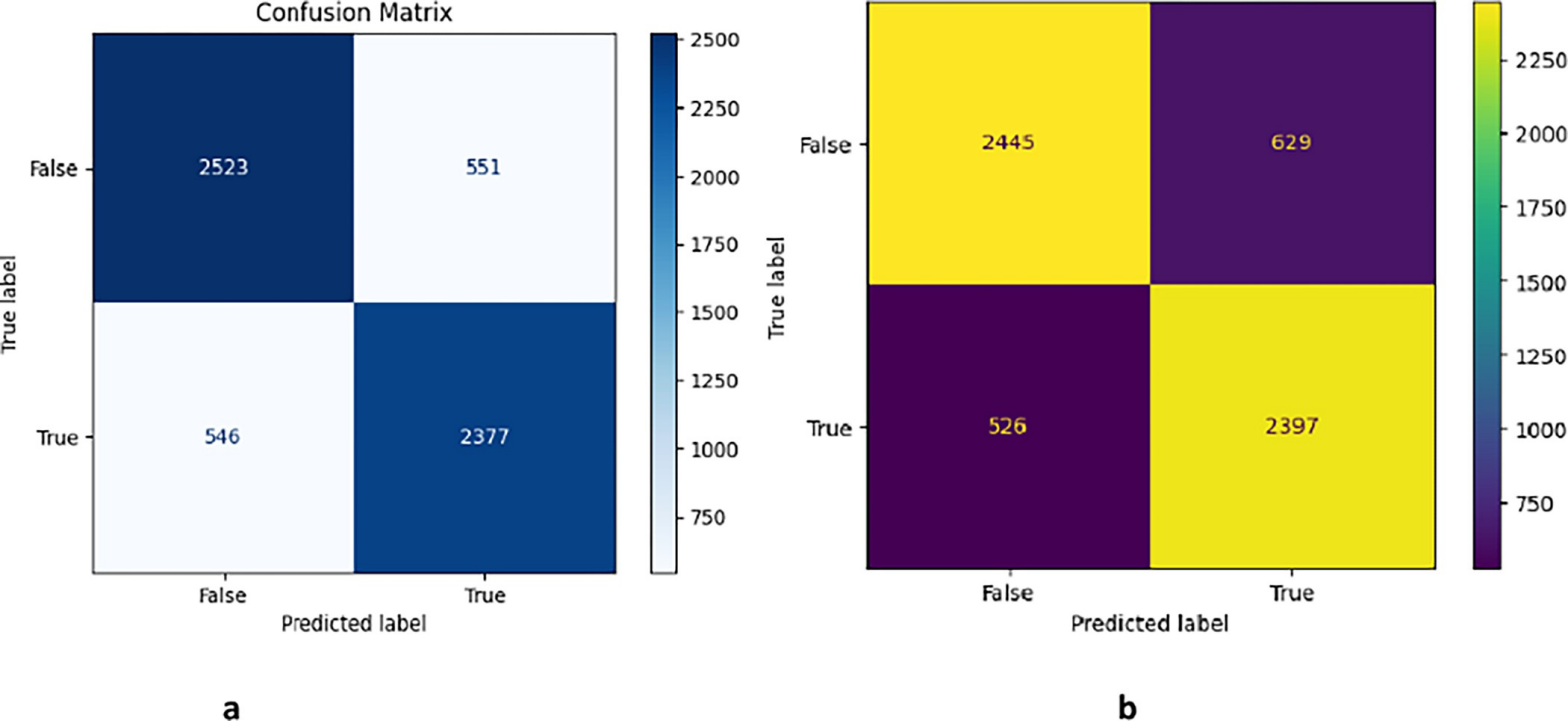
Confusion matrix generated for the ML classifier 4a for logistic regression and 4b for random forest.

The confusion matrix of the RF and LR classifier is shown in Fig 5, which is a way to visualize the performance of a classification model. In this case, the model is trying to classify comments as either sarcastic or not sarcastic. The rows of the matrix represent the true labels of the comments, and the columns represent the predicted labels. In the context of sarcasm detection, the most important metrics are likely to be precision and recall. Precision is the proportion of comments that were labelled as sarcastic that was sarcastic. Recall is the proportion of sarcastic comments that were correctly classified. In this case, the precision is 79%, and the recall is 82%. are relatively high, which suggests that the model is doing a good job of detecting sarcasm.

#### 5.1.5 Comparative analysis with state-of-art approach

**Fig 6:**
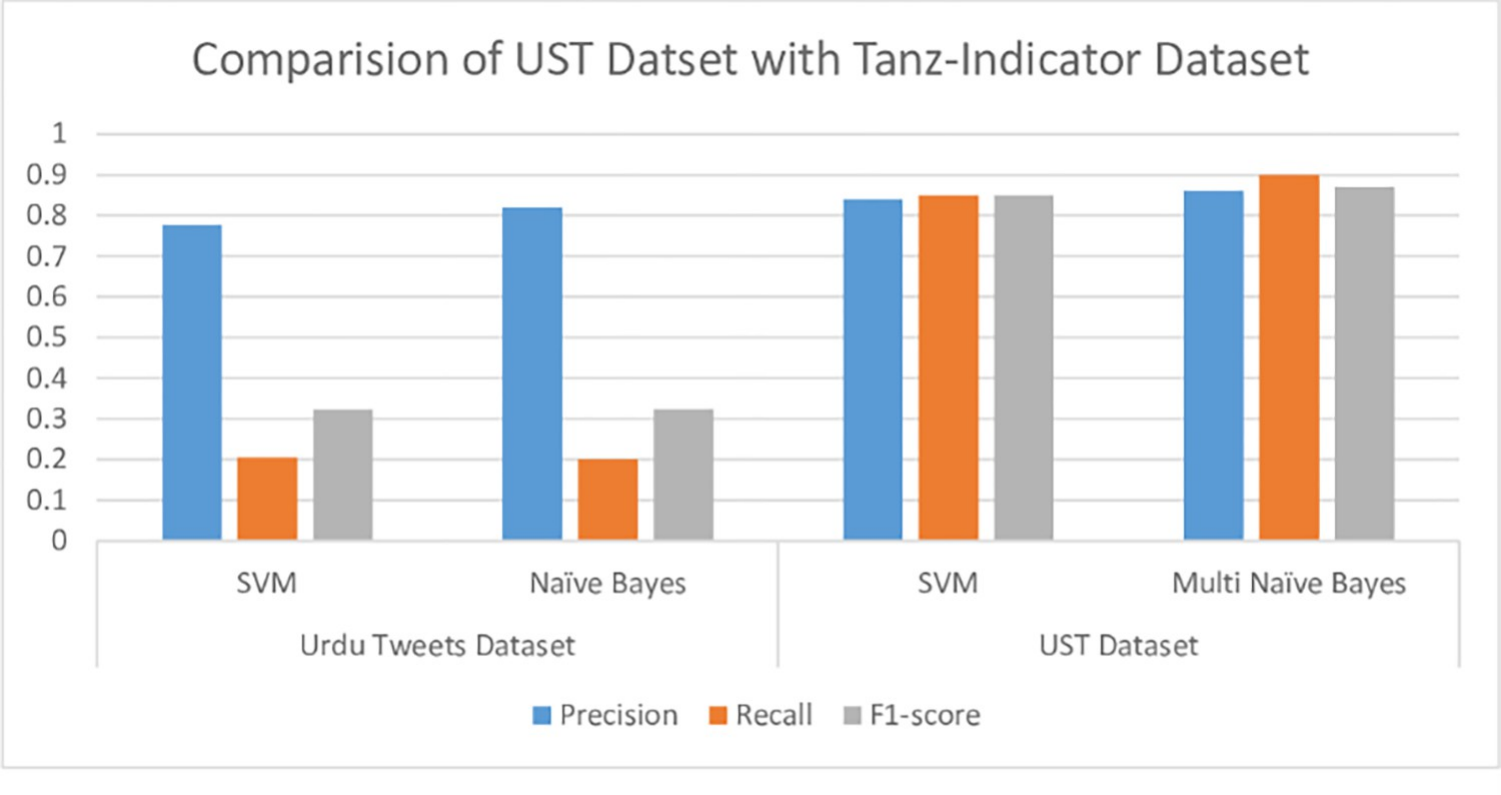
Performance comparison of classifiers on different datasets.

The proposed approach is compared with the previously published work Tanz-Indicator [16]. The Tanz-Indicator framework utilizes a rule-based to assess linguistic characteristics in Urdu sarcastic expressions. They employ Naïve Bayes and Support Vector Machine (SVM) classifiers to predict sarcasm in the Perso-Arabic script. The proposed framework adopts a feature-based Machine Learning (ML) approach, and secondly, we integrate ML techniques using textual features on the UST dataset. The paper presents a comprehensive analysis of performance metrics (Precision, Recall, F1-score) for different models applied to distinct datasets utilizing various sarcasm classification techniques, detailed in Table 7 and Fig 6.

**Table 7:** Tanz-Indicator model compared with the proposed model comparison.

Techniques	Dataset	Model	P	R	F1
**Gul et al.** [16] Tanz-Indicator	**Urdu Tweets Dataset**	SVM	0.777	0.204	0.323
		Naïve Bayes	0.82	0.201	0.324
**Proposed Model**	**UST Dataset**	SVM	0.80	0.89	0.84
		Multi Naïve Bayes	0.77	0.87	0.82

Fig 6 illustrates the learning curves for the RF, Multi NB, Bernoulli NB, LG, and DT classifiers. These curves demonstrate the evolution of performance metrics across training iterations, shedding light on how well the model generalizes and adapts to different classifiers. The performance of the models varies depending on the dataset used. The Tanz-Indicator dataset comprises 3000 tweets, divided into 70:30 for training and testing. In contrast, the UST dataset encompasses 20000 tweets, split into 80:20 for training and testing purposes. For the evaluation process, we employed a robust k-fold cross-validation approach to ensure the reliability of our results.

In the context of the UST Dataset, the findings indicate that Multi Naïve Bayes outperforms SVM in precision, recall, and F1-score. Conversely, the Tanz-Indicator dataset demonstrates competitive performance between SVM and Naïve Bayes, displaying slight differences in precision and recall.

This study underscores the significance of considering the dataset when selecting the appropriate algorithm. The Multi Naïve Bayes model consistently exhibits superior performance across both datasets compared to SVM. This can be attributed to Naïve Bayes’ adaptability, demonstrating strong performance in smaller datasets due to its ability to handle limited instances and scaling effectively to larger datasets owing to computational efficiency and enhanced learning from increased data volumes.

The comparative analysis with the Tanz-Indicator framework is pivotal in contextualizing the advancements made by our proposed approach. Tanz-Indicator, as a rule-based system, represents a foundational effort in Urdu sarcasm detection. However, the proposed ML methodology, particularly the SVM and Multi Naïve Bayes models, markedly outperforms Tanz-Indicator across all key metrics—precision, recall, and F1-score. This performance disparity underscores a fundamental shift in approach efficacy. While rule-based systems like Tanz-Indicator rely on predefined linguistic patterns, they may struggle with the fluidity and contextual nuances inherent in sarcastic Urdu expressions. In contrast, our ML models, trained on the extensive UST dataset, can capture these nuances more effectively. The higher F1-scores, 0.84 for SVM and 0.82 for Multi Naïve Bayes compared to Tanz-Indicator’s 0.323 and 0.324 reflect a balanced improvement in both precision and recall. This balance is crucial in sarcasm detection, where misclassifications can significantly impact sentiment analysis and user experience. The comparative analysis also highlights the scalability and adaptability of the proposed approach. By leveraging a larger, more diverse dataset UST with Tanz-Indicator’s dataset, our models demonstrate robustness in handling the variability of user-generated content. This scalability is vital for real-world applications where the volume and diversity of data are ever-increasing. The juxtaposition with Tanz-Indicator not only quantifies the performance gains of our ML-based approach but also qualitatively illustrates its superiority in capturing the linguistic intricacies of Urdu sarcasm. This comparison underscores the potential of data-driven, machine-learning methodologies in advancing NLP tasks for low-resource languages like Urdu, paving the way for more nuanced, context-aware sentiment analysis tools.

#### 5.1.6 Statistical significance

We conducted a paired t-test with a significance level (alpha) of 0.05 to assess the significance of the UST dataset. In this analysis, the one-tailed and two-tailed p-values are notably small (less than 0.05), indicating compelling evidence supporting the significance of the proposed ML model concerning the UST dataset. The results of the significant test are shown in Table 8.

**Table 8:** Significance of our UST dataset in terms of T-Pair test.

Baseline	Proposed	P(T ≤ t) Two-Tail	P(T ≤ t) One-Tail	Remarks
**Tanz-Indicator dataset**	**UST Dataset**	0.005431	0.010861	Highly significant

## 6. Discussion

This research explores the complex aspects of sarcasm detection in Urdu, a low-resource language with cultural nuances. Recognizing sarcasm is crucial in text categorization, significantly advances NLP research, and facilitates practical applications across diverse domains. The study’s objective is to exploit the instances of sarcasm within low-resource languages, necessitating understanding the linguistic nuances and cultural context. Sarcasm often relies on linguistic cues, tone, and contextual references, which vary significantly across languages. To fulfill the research objectives, we thoroughly evaluated various state-of-the-art ML models for sarcasm detection in Urdu, highlighting the significance of linguistic nuances and cultural references.

This research finding highlights the significant challenge of the scarcity of standard annotated datasets for recognizing sarcasm in low-resource languages like Urdu. Although automatic sarcasm detection has been widely researched in English compared to other languages particularly, low-resource languages like Urdu. To handle the dataset issue, we curated an Urdu-Sarcastic-Tweets-Dataset (UST) comprising user-generated comments from social media, including sarcastic and non-sarcastic statements in Urdu. The core objective is to provide open access to this dataset, encouraging other researchers to use it as a criterion testbed for sarcasm recognition. The data collection process itself exposed nuances specific to sarcasm in Urdu. The data collection from 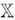 involves 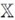 APIs widely used to extract tweets, especially those with hashtag sarcasm. However, there is no single most objective or standard data collection approach. While the hashtag for crawling on 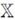 provides excellent insight for researchers. The labelled comments with hashtags present a clear data relationship, but the data quality is inaccurate. For example, ’I love walking. # not’. Here, the sarcasm is expressed through "#not." However, if the hashtag is removed, the statement ’I love walking’ may not be interpreted as sarcastic. Hence, the domain expert manually annotated most of the sarcastic datasets. Some well-known datasets for sarcasm detection include the Amazon product reviews dataset and Facebook comments. As per our study, there is a scarcity of standard sarcastic annotated datasets, particularly for low-resource language.

Prioritizing simplicity and clarity, we align with the suitability of fundamental ML algorithms for the proposed approach rather than relying solely on resource-intensive deep learning or transfer learning algorithms, which require vast corpora and substantial computational capacity. By focusing on simplicity and clarity, the proposed methodology ensures broader applicability and efficiency despite dataset limitations. The findings of our research have significant implications for both academia and practical applications. Improved sarcasm detection not only advances sentiment analysis but also enhances the functionality of chatbots, virtual assistants, and other NLP applications. Furthermore, our study sets the stage for future research in sarcasm detection for other low-resource languages, contributing to the broader goal of bridging language and cultural gaps in NLP.

The proposed approach offers several potential advantages regarding performance perspective, t. The integration of advanced ML models and sophisticated feature extraction techniques significantly enhances the accuracy of sarcasm detection in Urdu. Robust handling of linguistic nuances such as emojis and slang, coupled with effective data augmentation methods, contributes to the model’s improved performance and generalization capability. The scalability of our approach ensures its applicability to large datasets, making it suitable for real-world applications in sentiment analysis, social media monitoring, and human-computer interaction. These performance improvements not only advance the state of sarcasm detection in low-resource languages but also provide a foundation for cross-linguistic research and applications.

## 7. Conclusions

Identifying sarcasm in text is challenging, especially in languages like Urdu with limited linguistic resources. This research focused on a comprehensive approach to evaluate the performance of different ML classifiers in identifying sarcastic and non-sarcastic comments. Moreover, our research will delve into how contextual information and semantic analysis may be used to better comprehend the nuances of sarcasm in Urdu. The comparative analysis, juxtaposing uni-gram, bi-gram, and tri-gram models, provides valuable insights into the intricacies of feature extraction in natural language processing tasks. Notably, the uni-gram model emerged as the most effective, highlighting the importance of individual words in the Urdu language for sentiment analysis.

Recognizing the importance of data quality, we are committed to enhancing the UST dataset. Increasing its volume and incorporating language models can significantly boost the performance and accuracy of sarcasm detection methods. The ability to discern sarcasm in user communications holds immense potential, particularly in empowering applications like chatbots and virtual assistants. These advancements can foster more empathetic and context-aware interactions, driving progress in human-computer interactions and data analysis across various fields.

Advancements in sarcasm detection is to incorporate deep learning approaches, particularly the transformers approach. The transformer can capture complex relationships within sequential data, making them particularly well-suited for tasks that demand an understanding of nuanced language structures. Additionally, integrating fuzzy logic-based approaches can enhance our understanding of the subtle and context-dependent nature of sarcasm. These directions open avenues for more sophisticated models, promising improved accuracy and robustness in identifying sarcasm in Urdu.
